# Editorial: Predicting Individual Responses to Exercise Interventions

**DOI:** 10.3389/fphys.2020.559878

**Published:** 2020-12-23

**Authors:** Brendon Gurd, Giuseppe D'Antona, Vassilis Mougios

**Affiliations:** ^1^School of Kinesiology and Health Studies, Queen's University, Kingston, ON, Canada; ^2^Department of Public Health, Experimental and Forensic Medicine, CRIAMS-Sport Medicine Centre, Voghera, University of Pavia, Pavia, Italy; ^3^Laboratory of Evaluation of Human Biological Performance, School of Physical Education and Sport Science, Aristotle University of Thessaloniki, Thessaloniki, Greece

**Keywords:** exercise training, interindividual variability, genomics, proteomics, metabolomics

Despite the overwhelming evidence for the health benefits of regular exercise, there is limited knowledge regarding accurate prescription of exercise regimens on an individual basis, largely emanating from large interindividual differences in the biological responses to training. The present Research Topic has attempted to tackle this problem by gathering a sum of 14 papers attempting to discover predictors of the individual responses to exercise interventions.

Flück et al. report on potential association between gene polymorphism and performance in power and endurance elite athletes. In particular, by using an appropriate statistical approach, they tested whether gene polymorphism for angiotensin converting enzyme (ACE), tenascin-C (TNC), and actinin-3 correlates with diverse structural features of the skeletal muscle fibers known to contribute to the overall muscular function. Results indicated interaction effects between the athlete type and genotype for two gene polymorphisms of ACE (I/D) and TNC (A/T).

A similar approach was used by Ficek et al. to study the correlation between interleukin-15 (IL-15) polymorphism and training-induced changes in body composition in women subjected to a 12 week training programme. A significant interaction was found between genotype and changes in fat mass % (FM%) and fat-free mass (FFM). In particular, the genotype-phenotype interactions was robust for A allele and the [T;A] haplotype and FM% reduction and elevated FFM due to exercise.

The paper by Haun et al. deals with the identification of biomarkers in low and high previously well-trained responders to resistance training, clustered on the basis of changes in mean muscle fiber cross-sectional area (fCSA), muscle thickness, upper right leg lean soft tissue and mid-thigh circumference after 6 weeks of high-volume resistance training. Importantly, in this work regression analysis identified pre-exercise lower type II fiber percentage and fCSA as strong predictors of the hypertrophic response to resistance training.

The paper by Garai et al. deals with validity of the computer-based artificial neural network analysis (ANN) to predict individual responses to physical exercise. This approach is used to identify patterns of correlation between physiological and molecular datasets obtained in trained subjects in a concept-free manner. Using ANN, authors identified numerous molecular changes triggered by regular combined endurance-strength training in previously unaccustomed subjects and showed evidence for individual responsiveness at molecular level, identifying responders and not responders for each parameter.

Blume and Wolfarth sought potential performance-related predictors in young competitive athletes by monitoring 146 young athletes through ~10 months. The authors found that enhanced health senses and lower stress levels were related to performance progress. The authors propose that such subjective self-reported data should be added to the parameters routinely used to plan training, modify training intensity, and, ultimately, predict and ensure optimal performance development in young competitive athletes.

Ross et al. evaluated responses to exercise for health outcomes in overweight or obese adults at an individual level. By assigning 382 previously sedentary, overweight or obese individuals to either an inactive control group or to different exercise training programs, they found no association between changes in aerobic capacity and changes in visceral fat, the lipidemic profile or fasting plasma insulin. These findings underline the need for additional research on the biochemical, physiological, and genetics factors influencing individual responsiveness to exercise training regimens.

Tung et al. and Tung et al. explored physiological and biochemical differences between mice of intrinsically high and low exercise capacities (in terms of endurance and strength) through multiomics approaches. They report that, compared to the latter, the former displayed, on one hand, lower fatigue and injury biomarkers and, on the other hand, higher levels of muscle microRNAs associated with performance-related functions and key proteins related to muscle function and carbohydrate metabolism. Groups also differed in the gut microbiome.

O'Donoghue et al. examined whether phenotypic characteristics at baseline or phenotypic responses to a 12-week lifestyle intervention can explain inter-individual variability in change in glucose tolerance in 285 individuals at high risk of type 2 diabetes. Despite finding an overall improvement and despite performing a meticulous analysis, the authors found no sufficient phenotypic characteristics, among standard clinical and physiological parameters, that could explain the inter-individual variability in the response of glucose tolerance to the intervention.

The review by Petrigna et al. examined the reliability and feasibility of available procedures testing countermovement and squat jump in the context of public health examination in adolescence. By analyzing 117 relevant studies in the literature, the authors noted a lack of method standardization for both jumps. Based on the literature, they proposed standard operating procedures, which may facilitate the comparison of data between studies in testing lower limb muscle strength and power in adolescents.

Loro et al. examined differences in the activation of metabolic pathways within exercised skeletal muscle between control mice and mice lacking IL-15 receptor alpha (IL15RA). Mice lacking IL15RA demonstrated higher fatigue resistance, recovery capacity, and increased habitual activity compared to control mice. Skeletal muscle metabolomic analysis revealed 8 resting and 11 post-exercise differences in muscle metabolite content. These results provide some mechanistic insight into how IL15RA ablation improves exercise performance and highlight the potential impact of genetic variability on exercise response.

El Abed et al. studied changes in markers of oxidative stress in blood following sprint interval, endurance, and combined training in trained judokas. Although the comparison between exercise protocols yielded mixed results, the data do support the potential for differential oxidative stress responses following different intensities of exercise. These results provide a mechanistic bases for the importance of considering exercise intensity as we move toward personalized exercise prescription.

Sierra et al. examined differences in hemtological and iron metabolism responses between participants with/without the ACTN3 R577X polymorphism following a marathon race. Between genotype differences were not observed for fitness or for mean changes in hematological and iron metabolism responses. The proportion of participants with large changes in iron levels and hematological parameters was smaller following the marathon in athletes with the ACTH3 XX genotype. The results presented by Sierra et al. support the existence of a small genetic component in the determination of exercise response, however, future studies with larger samples and more rigorous statistical comparisons are needed.

Müllers et al. review the impact of physical activity on the development of dementia and explore the potential for personalized exercise prescription in the context of individual variability in response to exercise. The authors discuss the factors underlying inter-individual variability in response to exercise, the potential for individual patterns of response, and methods for ensuring benefit in most individuals. They conclude that more research is needed to expand our understanding of the potential for personalized exercise prescription. It seems particularly important for future work to determine if personalized exercise prescription can enhance cognitive responses to exercise and help to reduce/delay the onset of dementia.

He et al. examine the time course of myokine response to different exercise intensities. Higher intensity exercise increased peak post-exercise FGF-21 and follistatin but had no effect on IL-15, myostatin, irisin, resistin, or omentin. These data suggest that changes in submaximal intensity have inconsistent impact on the post-exercise myokine response. Whether individual variability exists in the acute myokine response to exercise is an interesting avenue for future research.

In conclusion, the articles in this Research Topic highlight the complexity of the factors determining individual responses to exercise interventions. We believe that, through both their positive and negative findings, they contribute to our understanding of the problem. Key improvements have been made by the discovery of new potential genotype-phenotype connections, as well as molecular, morphological and even psychological predictors of the responses to training. Future research on the topic should involve larger (and, possibly, multicenter) studies with sufficient statistical power to detect predictors of the responses to exercise interventions, along with an expansion of the spectrum of potential predictors in the areas of physiology, biochemistry, molecular biology, biomechanics, and psychology through interdisciplinary collaboration.

## Author's Note

We hope that the readers will enjoy, and benefit from, reading this collection. We welcome your feedback.

## Author Contributions

All authors have written and reviewed the editorial.

## Conflict of Interest

The authors declare that the research was conducted in the absence of any commercial or financial relationships that could be construed as a potential conflict of interest.

